# Evaluation of a blood miRNA/mRNA signature to follow-up Lu-PRRT therapy for G1/G2 intestinal neuroendocrine tumors

**DOI:** 10.3389/fendo.2024.1385079

**Published:** 2024-06-14

**Authors:** Virginie Jacques, Lawrence Dierickx, Jean Sebastien Texier, Severine Brillouet, Frederic Courbon, Rosine Guimbaud, Lavinia Vija, Frederique Savagner

**Affiliations:** ^1^ Biochemistry Laboratory, Federative Institute of Biology, Academic Hospital, Toulouse, France; ^2^ Inserm UMR1297, Institute of Cardiovascular and Metabolic Diseases, Toulouse, France; ^3^ Faculté de Santé, University Paul sabatier, Toulouse, France; ^4^ Nuclear Medicine Department, Regional Center of Cancer Care Oncopole Claudius Regaud, Toulouse, France; ^5^ Digestive Oncology Department, Academic Hospital, Toulouse, France

**Keywords:** intestinal neuroendocrine tumors, miRNA/mRNA signature, PRRT therapy, outcome, hematotoxicity

## Abstract

**Background:**

^177^Lu-oxodotreotide peptide receptor therapy (LuPRRT) is an efficient treatment for midgut neuroendocrine tumors (NETs) of variable radiological response. Several clinical, biological, and imaging parameters may be used to establish a relative disease prognosis but none is able to predict early efficacy or toxicities. We investigated expression levels for mRNA and miRNA involved in radiosensitivity and tumor progression searching for correlations related to patient outcome during LuPRRT therapy.

**Methods:**

Thirty-five patients received LuPRRT for G1/G2 midgut NETs between May 2019 and September 2021. Peripheral blood samples were collected prior to irradiation, before and 48 h after the second and the fourth LuPRRT, and at 6-month follow-up. Multiple regression analyses and Pearson correlations were performed to identify the miRNA/mRNA signature that will best predict response to LuPRRT.

**Results:**

Focusing on four mRNAs and three miRNAs, we identified a miRNA/mRNA signature enabling the early identification of responders to LuPRRT with significant reduced miRNA/mRNA expression after the first LuPRRT administration for patients with progressive disease at 1 year (*p* < 0.001). The relevance of this signature was reinforced by studying its evolution up to 6 months post-LuPRRT. Moreover, nadir absolute lymphocyte count within the first 2 months after the first LuPRRT administration was significantly related to low miRNA/mRNA expression level (*p* < 0.05) for patients with progressive disease.

**Conclusion:**

We present a pilot study exploring a miRNA/mRNA signature that correlates with early hematologic toxicity and therapeutic response 12 months following LuPRRT. This signature will be tested prospectively in a larger series of patients.

## Introduction

The incidence rates of neuroendocrine tumors (NETs) of the gastrointestinal tract vary between 0.9 and 9.97/100,000 around the world with an annual incidence of digestive NETs in France of at least 2.06 to 7/100,000 (1–5). Most gastrointestinal neuroendocrine tumors (GI-NETs) are well-differentiated and represent the second most prevalent digestive tumor after colorectal cancer. While the incidence of midgut NETs is increasing, the therapeutic options are limited when they become progressive, metastatic, or non-operable (1, 6). ^177^Lu-oxodotreotide or ^177^Lu-DOTATATE^®^ peptide receptor therapy (LuPRRT) targeting somatostatin receptors (SSTRs) is a molecular radiotherapy approach based on ^177^Lu irradiation. In the NETTER-1 phase III randomized clinical trial, ^177^Lu-DOTATATE proved its superiority compared to high-dose somatostatin analogs, as it dramatically increased progression-free survival (PFS) with a ~80% reduction in the estimated risk of tumor progression or death (7, 8). Although ^177^Lu-DOTATATE has been approved by the EMA as a first- or second-line treatment for G1/G2 non-operable metastatic GI-NETs, radiological responses were variable and 5-year overall survival (OS) was not significantly increased (9).

The risk of severe short-term hematologic toxicities (mainly thrombocytopenia, lymphopenia, and anemia) is low (25%), even though persistent lymphopenia has been described as potentially compromising other systemic therapeutic modalities. LuPRRT may also expose patients to irreversible late toxicities such as lymphoproliferative disorders, especially in those likely to have improved OS (7). Unlike for external beam radiotherapy (EBR), dosimetry for LuPRRT lacks accuracy, thereby hampering dose–effect correlation studies and the prediction of late toxicity (10). Similarly to how genetic environment predicts the late toxicity after EBR, lymphocytes expressing SSTR may also reflect the radiosensitivity to LuPRRT irradiation (11, 12). ^177^Lu-DOTATATE administration follows a fixed therapeutic scheme using the same activity of ^177^Lu-DOTATATE per cycle and patient, even though different patients have different responses and tolerance profiles. Therefore, there is a pressing need to be able to forecast and identify the most suitable patients and the right timing for LuPRRT therapy to obtain long-term disease control avoiding hematotoxicity or irreversible side effects such as myelodysplastic syndrome (MDS).

Various clinical parameters such as tumor grading and associated comorbidities, general biomarkers [such as chromogranin A (CgA) and 5-hydroxyindolacetic acid (5HIAA)], and imaging criteria (RECIST 1.1 criterion for CT and MRI) have already been investigated and may be used to establish a relative disease prognosis, but none is able to predict late toxicity or the outcome of LuPRRT. Recently, a test based on peripheral blood transcript analysis (NETest, PPQ) showed promising results for predicting LuPRRT efficacy (10, 13, 14). However, it is not yet available in routine practice, and it does not provide correlations with radiosensitivity or toxicities limiting treatment continuation (15). As miRNAs are master modulators of gene expression, miRNA/mRNA combinatory profiles could provide new information on the mechanisms underlying therapeutic efficacy. Previous studies of the sole quantification of miRNAs in the blood have shown that few of them could be proposed as prognostic or predictive biomarkers in GI-NET (16, 17). A major step forward would be to hone these profiles to investigate specific molecular pathways, especially those in heterogeneous metastatic tumors such as GI-NETs (18).

We hypothesize that an integrative approach combining the expression of mRNA and miRNA in genes highly responsive to irradiation and involved in tumor progression could lead to a specific gene expression signature from peripheral blood to predict patient outcome and the risk of hematologic toxicity during LuPRRT treatment.

## Materials and methods

### Patients

A total of 35 patients were treated with LuPRRT for G1 or G2 NET between May 2019 and September 2021. All participants provided written informed consent for LuPRRT and molecular genomic analysis (clinical trial GENEBIOLuNET, NCT03667092). Treatment consisted of four IV administrations of 7400 MBq Lu-177 DOTATATE^®^ at 2-month intervals. Objective response rate was assessed using RECIST 1.1 criteria and defined as either responder in terms of disease control (partial or complete response or stabilization) or non-responder (progressive disease) (7). Patients who progressed or died during LuPRRT treatment or follow-up were included. Follow-up and OS were assessed starting from the first day of LuPRRT administration.

For expression analysis, seven peripheral blood samples on PAXgene^®^ tubes were collected: prior to irradiation (P1 and P2), before and 48 h after the second (P3 and P4) and the fourth (P5 and P6) LuPRRT administration, and at 6-month follow-up (P7). The first two samples (P1 and P2) were performed at 48-h intervals prior to the first LuPRRT administration, with the second sample obtained 2 h before starting amino acid perfusion, to test time-related variation in gene expression independently from LuPRRT treatment. Samples were stored in the CRB-TBR of the University Hospital of Toulouse (Collection number: DC-2015–2450).

For plasma CgA and urinary 5HIAA assays, three blood or urinary samples were collected from each patient as such: prior to irradiation (P1) and before the second (P3) and the fourth (P5) LuPRRT administration. CgA was measured using BRAMHS CgA II on a Kryptor apparatus. A positive CgA is >102 ng/mL. The 24-h urinary 5HIAA was measured using an LCMS-MS apparatus (Shimadzu, LCMS-8060) and considered negative when <40 µmol/24 h. Grade 1 NET was defined as Ki-67 index <3% and mitotic rate <2, whereas grade 2 was defined as Ki-67 index = 3%–20% and mitotic index = 2–20 (19). Performance status was assessed by ECOG classification. The absolute lymphocyte count (ALC) NADIR was determined on blood collected just before treatment and then 15 (NADIR1) and 45 (NADIRC2) days after the first LuPRRT administration. Lymphopenia was classified using the CTCAE criteria (Common Terminology Criteria for Adverse Events, version 5.0.).

### miRNA and mRNA extraction, quantification, and profiling

Blood samples on PAXgene^®^ were kept at +4°C for up to 5 days and then frozen at −20°C. mRNA and miRNA were extracted using the PAXgene^®^ blood miRNA kit (Qiagen) and quantified using a Nanodrop apparatus (ThermoScientific Fisher Scientific). After retrotranscription, expression was quantified using the SYBRgreen reagent on a Light cycler 480 apparatus. We used the All-in-One miRNA qRT-PCR detection kit (Qiagen) for miRNA and Superscript Vilo and Mastermix Sybregreen^®^ (Thermofisher Fisher Scientific) for mRNA with appropriate primers according to the manufacturer’s recommendations (**Supplementary Table 1**).

The expression of 13 genes was explored and was correlated with tumor aggressiveness (SSTR3 and 5), proliferation (BRAF-proto-oncogene), metabolism (ATP6V1H, PANK2, and HDAC9), signaling modulation (CXCL14), post-radiation DNA repair (XPC and DDB2), cell cycle (CDKN2A and CDKN1B), and apoptosis signaling (BAX) with the normalization gene GAPDH. A panel of six miRNAs (miR-31, miR-129–5p, miR-133a, miR-215, miR-196b, and miR-183) was also explored and normalized to RNU6–1. Clinical studies have shown that these miRNAs were dysregulated in both tumor and peripheral blood compartments of patients with GI-NET (12, 13). All these genes and miRNAs have been independently described in the literature as related to GI-NET outcome. mRNA/miRNA combinations related to DNA repairing pathway, as well as their modulations on follow-up could be used to improve LuPRRT (20).

The fold change in gene expression was calculated for each sample using the 2^−^ΔΔ^Ct^ method. We first used quantile normalization for mRNA and miRNA profiles and *Z*-score normalization to combine miRNA and mRNA expression and identify signatures for all (12 mRNA and 6 miRNA) and the most relevant (4 mRNA and 3 miRNA) genes. Multiple regression analyses were performed to identify a combined miRNA/mRNA signature that would best predict the response to LuPRRT and hematologic toxicity.

### Statistical analysis

We performed a Mann–Whitney test for quantitative variables for comparison between groups and one-way ANOVA to compare different time points for the same patient. Multivariable analyses were performed using the Cox proportional hazard model to study the influence of biomarkers on time-to-event outcomes after adjusting for prognostic factors. We estimated OS using the Kaplan–Meier method. No evidence of confusing factor was noticed regarding age, sex, and previous treatment. *p < 0.05, ** *p* < 0.01, ***p < 0.001, ****p < 0.0001.

## Results

### Patients

A total of 35 patients (21 men and 14 women) were treated with Lu-177 DOTATATE between May 2019 and September 2021 and had peripheral blood sampled for mRNA and miRNA during the study. A total of 28 patients presented with midgut NETs of the small intestine, 3 patients had a NET within the right colon, 3 in the ileo-cecal valve, and 1 in the appendix (**Table 1**). A total of 31 patients received four Lu-177 DOTATATE injections, 1 patient died after the first LuPRRT, 1 died after the second LuPRRT, 1 patient refused the fourth injection, and 1 developed irreversible grade 2 thrombocytopenia and stopped treatment after the third injection. One-year follow-up radiological assessment using the RECIST 1.1 criteria was possible in 32 patients (3 died before): 3 patients had partial response at 1-year follow-up and 24 patients were stable; thus, 27/35 patients (78%) were considered as responders as disease was controlled. Five patients had progressed. Radiological follow-up at 2 years was possible for 29 patients as 6 died before 24 months: 7 patients progressed and 22 patients (63%) were stable and considered as responders (**Table 2**). Only three patients underwent FDG PET.

**Table 1:** Baseline clinical characteristics of patients.

Characteristics		Data	
***Sex, n (%)***	Male	21 (60)	
	Female	14 (40)	
Age, years			
	**All patients**	67 [52;83]	
	Male	71[56;83]	
	Female	65 [52;72]	
***Body mass index***		24.04 [16.8; 37.18]	
***Median time (months) since diagnosis***		52	
Primary tumor site, n (%)			
	Ileum	19 (54)	
	Jejunum	1 (3)	
	Midgut	6 (17)	
	Small intestine	2 (6)	
	Right colon	3 (9)	
	Ileo-cecal valve	3 (9)	
	Appendix	1 (3)	
Site of metastasis, n (%)			
	Liver	32 (91)	
	Lymph nodes	29 (83)	
	Mesentery	23 (65)	
	Bone	17 (49)	
	Lungs	1 (3)	
	Ovaries	1 (3)	
	Mammary gland	1 (3)	
	Other	5 (14)	
Ki-67 index			
	0%–2%	12 (34)	
	3%–20%	23 (66)	
Previous treatments			
	Somatostatin analogs	31 (89)	
	Surgery	22 (63)	
	Everolimus	3 (9)	
	Chemotherapy	6 (18)	
	Locoregional therapy	3 (9)	
***Performance status (ECOG)***	0	18 (51)	
	1	14 (40)	
	2	3 (9)	
	3–4	0	
***Somatostatin analogs during PRRT***	No	7 (20)	
	Yes	28 (80)	

Qualitative data are expressed as numbers (n) followed by percentages in brackets; continuous data are expressed as mean ± SD.

**Table 2:** Objective tumor response.

	12 months	24 months
***Complete response, n (%)***	0	0
***Partial response, n (%)***	3 (9)	0
***Stability, n (%)***	24 (69)	22 (63)
***Progression, n (%)***	5 (14)	7 (20)
***Not known as patient died before imaging***	3 (9)	6 (17)
***Non-responders***	8 (23)	13 (37)

Objective response at 12 and 24 months defined as a response according to Response Evaluation Criteria in Solid Tumors (RECIST 1.1).

Of the 35 patients explored in this study, 18 (51%) had grade 1–2 lymphopenia and 7 (20%) had grade 3 lymphopenia on 15 days after the first injection of LuPRRT. In the global follow-up, 11 (31%) patients presented with grade 1–2 lymphopenia, 21 (60%) presented with grade 3 lymphopenia, and 3 (9%) presented with grade 4 lymphopenia.

### Gene expression stability and kinetics during PRRT treatment

**Figure 1:**
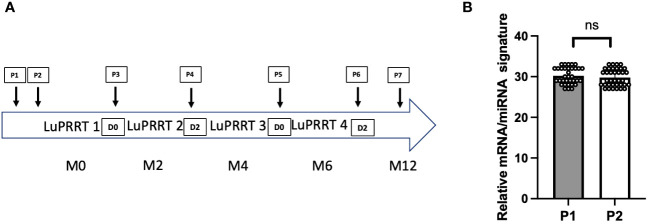
Overview of the study. **(A)** Presentation of the blood samples timing within the LuPRRT treatment frame. **(B)** Gene expression stability before LuPRRT therapy: difference between two different blood samples (P1 and P2) obtained at a 48-h interval before starting LuPRRT (*p* = 0.68). Non-significant (ns).

In 35 patients with serial blood samples collected during LuPRRT treatment (from P1 to P7 according to **Figure 1A**), first two samples were collected before the first LuPRRT injection with an interval of 48 h (P1 and P2) to explore the stability of a signature including 12 mRNAs and 6 miRNAs. The variation in miRNA/mRNA signature during this 48-h period was non-significant, thus confirming the stable expression of these genes prior to LuPRRT treatment (**Figure 1B**; ns; *p* = 0.68).

**Figure 2:**
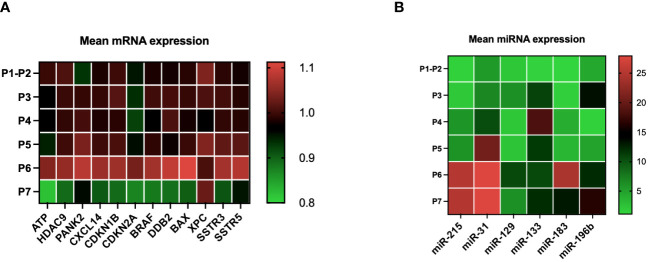
mRNA and miRNA expression kinetics during LuPRRT: before (P1–P2) treatment, before and after second LupRRT (P3 and P4), before and after the fourth LuPRRT (P5 and P6), and at 6-month follow-up (P7) **(A)** Variation in the expression of 12 genes (mRNA) relative to those of GAPDH. **(B)** Variation in the expression of six miRNAs relative to those of RNU6–1.

The evolution of the mean expression profile of various genes for all patients treated with LuPRRT (P3 to P7) was measured before and 48 h after the second (P3 and P4) and the fourth (P5 and P6) LuPRRT as well as 6 months later (P7) in order to identify the best combination of mRNA and miRNA related to patient outcome. miRNA expression levels were more differentially expressed during LuPRRT treatment compared to those of mRNA with an inverse expression pattern in relation with the intricate and dynamic nature of gene regulation (**Figures 2A, B**). The expression of SSTR3 and SSTR5 was used to confirm SSTR targeting throughout LuPRRT treatment with high expression levels at P1–P2 compared to other genes. Searching for a predictive signature, all candidate mRNAs and miRNAs were subjected to a stepwise multivariate Cox’s model, resulting in a total of four mRNA and three miRNA selection that we used as the final signature exploring damaged DNA repair (XPC and DDB2), regulation of cell proliferation and autophagy (BAX and BRAF), lymphocyte B/T activation (miR-31 and miR-133), and regulation of angiogenesis and tumor suppression (miR-196b) (**Supplementary Table 2**). We have identified early biomarkers of LuPRRT response as BAX, DDB2, miR-133, and miR-31, suggesting ongoing radiobiological effects while XPC, BRAF, and miR-196b biomarkers presented continuous variation during LuPRRT treatment, suggesting sustained activation of antitumor cellular pathways as a result of ^177^Lu irradiation.

### Biomarkers and outcome

**Figure 3:**
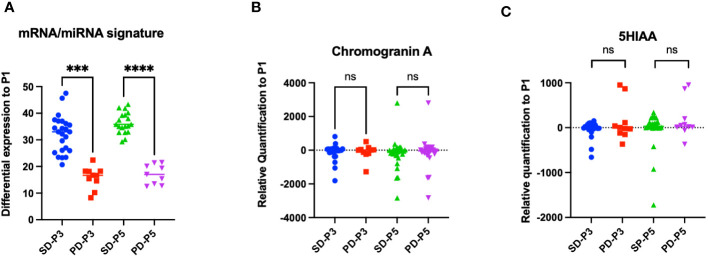
Differential profiles before and 48 h after the second and the fourth LuPRRT injection for miRNA/mRNA signature, chromogranin A, and 5HIAA for patients with stable disease (SD) or progressive disease (PD) at 12-month follow-up. **(A)** miRNA/mRNA signature before the second (P3) and fourth (P5) LuPRRT administration for four mRNAs and three miRNAs in patients considered as responders (SD-P3 and SD-P5) versus progressive patients (PD-P3 and PD-P5). **(B)** Chromogranin A levels before the second (P3) and fourth (P5) LuPRRT administration in responders (SD) or RECIST progressive (PD) patients at 12-month follow-up (*p* = 0.65). **(C)** 5HIAA levels before second (P3) and fourth (P5) LuPRRT administration in responders (SD) or RECIST progressive (PD) patients at 12-month follow-up (*p* = 0.24). ***p < 0.001, ****p < 0.0001; ns, non-significant.

We compared the miRNA/mRNA signature from blood samples obtained before the second (P3) and the fourth (P5) administration of LuPRRT relative to blood samples obtained before starting LuPRRT (P1–P2). We considered patients as responders (SD for stability or partial response) and non-responders [progressive disease (PD)] according to RECIST criteria on CT/MRI performed at 12 months of follow-up (**Figure 3A**). We observed that patients with PD 12 months after the last cycle of PRRT (*n* = 5 patients) presented significantly low levels of miRNA/mRNA expression just after the first LuPRRT administration (PD-P3) compared to patients with stable disease or partial response (SD-P3) (**Figure 3A**, *p* < 0.001). These differences were even more significant for samples collected 2 months after the third LuPRRT injection (PD-P5 vs. SD-P5), emphasizing the relevance of the signature (*p* < 0.001).

The differential expression levels of biomarkers currently used for GI-NET such as plasma CgA and urinary 5HIAA were dynamically assessed before each LuPRRT. CgA is a general NET biomarker whereas 5HIAA is a functioning marker mainly used for diagnosis and follow-up. We compared the variation at 2 months after the first injection of LuPRRT (P3) as well as after the third LuPRRT administration (P5) versus baseline in responders versus non-responders at 12-month follow-up (**Figures 3B, C**). There was no significant difference in CgA and 5HIAA during LuPRRT between responders (SD) or non-responders (PD) according to RECIST criteria (*p* = 0.51 and 0.24, respectively).

Multivariate analysis showed that patient outcome could be predicted by miRNA/mRNA expression profiles after the first (*p* = 0.0064) and the third LuPRRT (*p* = 0.0105), whereas Ki-67 index was at the limit of significance (*p* = 0.0521) (**Table 3**). We finally explored the OS (interquartile range) of the 35 patients according to the Ki-67 index. At the time of analysis, median OS was not reached and patients with a Ki-67 index greater than or equal to 10 (*n* = 9, 25%) showed a non-significant trend towards poor outcome (**Supplementary Figure 2**).

**Table 3:** Multivariate analysis of clinical and biologic parameters and miRNA/mRNA signature.

Variable	p-value	
***miRNA/mRNA signature at P3***	0.0064	*
***miRNA/mRNA signature at P5***	0.0105	*
***NADIR C1***	0.2501	ns
***NADIR C2***	0.4285	ns
***5HIAA at P3***	0.2773	ns
***5HIAA at P5***	0.4446	ns
***CgA at P3***	0.1933	ns
***CgA at P5***	0.1346	ns
***KI-67 index***	0.0521	ns
***RECIST12m***	0.0237	*

Multivariate analysis. *p < 0.05; ns, non-significant.

### Expression profile and hematologic toxicities

To test the potential correlations between our miRNA/mRNA signature and early hematological toxicity, we monitored the lymphocyte count at 15 and 45 days after LuPRRT administration. We considered as NADIR the lowest point of ALC after LuPRRT cycle 1 (NADIRC1) and 2 (NADIRC2) as indicators of the immune response. ALC nadir 15 days after the first LuPRRT administration (NADIRC1) was significantly related to low miRNA/mRNA signature at the time of the second (PD-P3; *p* = 0.036; *r* = −0.43) and fourth injection (PD-P5; *p* = 0.004; *r* = −0.63) for patients with PD. The same trend was also noticed for ALC nadir 45 days after the first LuPRRT (NADIRC2), suggesting that lymphopenia might be associated with radiosensitivity in responders to LuPRRT (**Supplementary Figure 2**).

**Figure 4:**
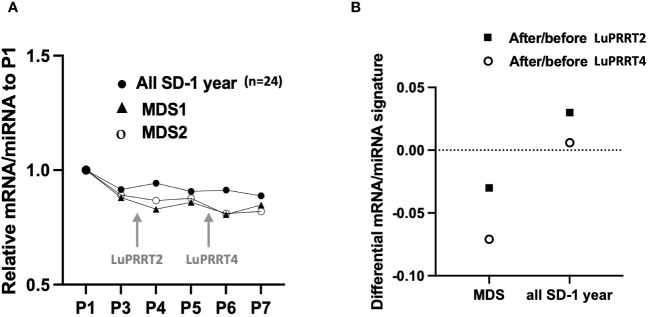
miRNA/mRNA signature for two patients with myelodysplasia (MDS) compared to all patients with stable disease at 1 year follow-up (SD, *n* = 24). **(A)** Kinetics of miRNA/mRNA signature during LuPRRT treatment for each patient presenting with MDS. **(B)** Variations of miRNA/mRNA signature at the second and fourth LuPRRT injection using the after/before LuPRRT signature ratio.

For two patients with stable disease at 1-year follow-up (SD), we identified a significant decrease in the miRNA/mRNA signature compared to other patients of the group (*n* = 24) after the second (P4; *p* < 0.05) and the fourth injection (P6; *p* < 0.05) (**Figure 4A**). That difference was emphasized when comparing miRNA/mRNA signature directly before and after the second (P4 vs. P3) and the fourth (P6 vs. P5) LuPRRT injection (**Figure 4B**). One patient developed grade 3 thrombocytopenia associated with grade 3 lymphopenia and grade 1 anemia. Several myelograms have been performed and a diagnostic of therapy-related MDS was confirmed 2 years after LuPRRT. This patient remained stable on RECIST criteria at 3-year follow-up. Another patient developed MDS with pancytopenia diagnosed 1 month after the fourth LuPRRT administration. The latter patient had stable disease until he died from therapy-related MDS 1 year after LuPRRT.

## Discussion

The clinical utility of circulating biomarkers to propose miRNA or mRNA profiles related to diagnosis, prognosis, and therapeutic targets has been often discussed independently (16, 17, 21). The NETest, exploring the expression of 51 genes (mRNA) with the aid of four prediction algorithms, led to define a score proportional to disease activity (22, 23). However, depending on the cutoff values, the specificity of this test varies among independent studies showing the difficulties to standardize mRNA expression profiles (24–26). The NETest, in combination with Ki-67, has been proposed to identify responsive tumors by defining a PRRT predictive quotient (PPQ) (10). Because of their stability and their regulatory role, changes in miRNA expression could also be associated to prognosis and therapeutic efficacy (18, 27, 28). In our study, we have combined mRNA expression to miRNA profiles to focus on an accurate response signature to LuPRRT. Each patient has been used as its own control using stable expression signature before LuPRRT treatment for standardization. Half of our selected mRNAs (BAX, XPC, DDB2, CXCL14, CDKN2A, and CDKN2B) are independent of the NETest signature but were described in the literature as related to radiosensitivity or DNA damage (10, 13–15, 29). The miRNAs we analyzed are also biomarkers of radiosensitivity as previously described (30–32). These miRNAs had shown a particular expression in metastatic NETs and stability under somatostatin analog therapy. Moreover, each biomarker of our panel was described as dysregulated in both the tumor and peripheral blood compartments for patients with GI-NET, suggesting their specificity (33). We reveal on this preliminary study that combining mRNA and miRNA expression profiling could be an interesting option to use in order to assess patient sensitivity and outcome to LuPRRT as previously revealed for the diagnosis and prognosis of several cancers (34–36). Our miRNA/mRNA signature focusing on a combination of four mRNAs and three miRNAs should be able to detect the LuPRRT responders earlier than NETest. This miRNA/mRNA signature appears to be able to detect the outcome of patients as early as the first administration of LuPRRT, allowing possible therapeutic adaptation, whereas the PPQ score derived from the NETest shows a significant difference only after four cycles of PRRT treatment (10). However, these results should be confirmed in a larger series due to the small sampling of our study.

In our cohort, only three patients underwent FDG PET, and it was not relevant to confront FDG PET status with our signature in this study. However, ENETS guidelines for colorectal NETs recommend FDG PET only in metastatic non-operable high-grade and G3 NETs, and the utility of FDG PET in the management of metastatic NET remains controversial (37, 38).

In the context of LuPRRT, studies have shown that patients with GI-NETs and a high Ki-67 expression are less likely to respond to treatment and may have a shorter PFS and OS (39). In our study, patients with a Ki-67 index of less than 10% had a higher rate of response to LuPRRT on the miRNA/mRNA signature but no significant difference in OS to those with a Ki-67 index of 10% or higher. According to previous recommendations (40), we confirm that merging Ki-67 index and expression signature is a useful prognostic factor for predicting stable disease and OS in response to PRRT efficacy with a higher predictive value of targeted miRNA/mRNA signature compared to Ki-67.

A total of 25 (71%) patients treated with LuPRRT presented lymphopenia with an early nadir 15 days after therapy and a subsequent slow partial recovery. This haematologic toxicity is related to LuPRRT and not influenced by previous chemotherapies (41). Lymphopenia observed during LuPRRT mainly affects the B-cell subpopulation and could be due to the therapeutic sensitivity of cells expressing SSTR (42). The ALC nadir can provide information about a patient’s radiosensitivity. Several studies have shown that ALC nadir is associated with a better response to LuPRRT treatment and improved OS in patients with GI-NETs (42, 43). Patients with GI-NET who had an ALC nadir of less than 0.5 × 10^9^/L had a significantly higher OS rate than those with an ALC nadir of 0.5 × 10^9^/L or higher. We thus identified a significant inverse relationship between miRNA/mRNA signature after the first PRRT cycle and the ALC nadir. Moreover, two patients who developed a LuPRRT-related MDS had a remarkable decrease in their miRNA/mRNA signature. This signature might therefore reveal the risk of increased clonal hematopoiesis or the risk of developing an MDS as soon as after the first LuPRRT perfusion, thus limiting the pursuit of the treatment. miRNA/mRNA profiling could indicate not only NET lesions’ radiosensitivity but also lymphocytes’ radiosensitivity and could be compared to the quantification of γ-H2AX foci in lymphocytes and absorbed dose to tumor and bone marrow (12).

LuPRRT is a promising treatment for patients with GI-NETs, although 15%–20% of patients show disease progression as early as 6 months post-PRRT (8, 44). We have explored in a pilot study a miRNA/mRNA signature that could predict patient outcome with a significant down-expression profile after the first cycle of LuPRRT in patients presenting with PD 12 months after therapy. This miRNA/mRNA signature is superior to CgA and 5HIAA assays as they reflect variations related not only to early response to LuPRRT therapy but also to hematologic toxicity. Further research is required to validate these findings in a larger series of patients in a multicenter setting in order to determine the clinical utility of this miRNA/mRNA profiling in guiding LuPRRT treatment decisions.

## Data availability statement

The original contributions presented in the study are included in the article/supplementary material, further inquiries can be directed to the corresponding author/s.

## Ethics statement

The studies involving humans were approved by Ethical Review Board From The South East I President: M Philippe RUSH Vice President: M Francois Falsan Secretary M Maurice MINAIRE Committee date of meeting 05/07/2021 Reference No ERB 2018-46 Project Research project involving human beings, interventional with only minimal risks and constraints, according to the list from December the 2nd 2016 (Article L.1121-1, 2 CSP) Received on 17/06/2021 Substantial modifications Amendment no 2 Documents concerned Protocol Version 4.0 08/06/2021 Protocol Resume Version 4.0 08/06/2021 Letter describing the modifications NO EudraCT or ID RCB 2018-001399-39 REF Promoter RC31/17/0356 Project Title: Pilot study for measuring molecular biomarkers and their capacity to characterize radionuclide therapy with Lu-177 DOTATATE) in metastatic G1-G2 neuroendocrine midgut tumors Acronym: GENEBIOLuNET Promoter: CHU TOULOUSE. The studies were conducted in accordance with the local legislation and institutional requirements. The participants provided their written informed consent to participate in this study.

## Author contributions

VJ: Writing – review & editing, Writing – original draft. LD: Investigation, Data curation, Writing – review & editing. JT: Investigation, Writing – review & editing. SB: Data curation, Writing – review & editing. FC: Writing – review & editing. RG: Validation, Data curation, Writing – review & editing. LV: Funding acquisition, Data curation, Writing – review & editing, Writing – original draft. FS: Project administration, Methodology, Funding acquisition, Writing – review & editing, Writing – original draft.
