# Supplementary material for: Evaluation of a blood miRNA/mRNA signature to follow-up Lu-PRRT therapy for G1/G2 intestinal neuroendocrine tumors
Source: Front Endocrinol (Lausanne). 2024 Jun 14;15:1385079. doi: 10.3389/fendo.2024.1385079 (PMC11212830; doi:10.3389/fendo.2024.1385079)
Supplement: Supplementary file 1 [file DataSheet_1.pdf]

Supplemental data

Figure S1: Overall survival relative to KI-67 index

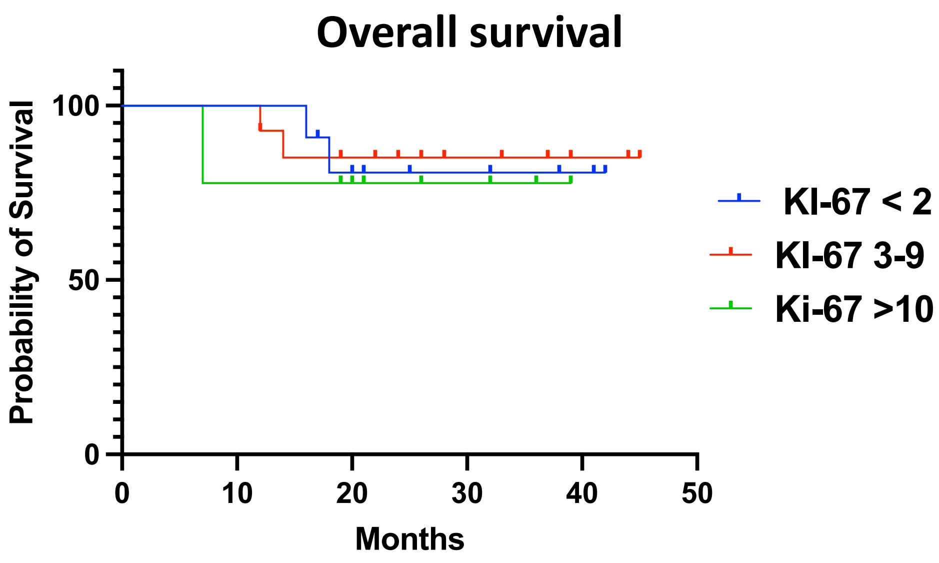

Figure S2: Pearson correlation 2x2 contingency table for lymphopenia at 15 days post first LuPRRT administration (NADIRC1) versus nadir at 45 days post first Lu PRRT administration (NADIRC2), Ki-67, and miRNA/mRNA signature after first LuPRRT in responder (SD-P3 and SD-P5) versus progressive patients (PD-P3 and PD-P5).

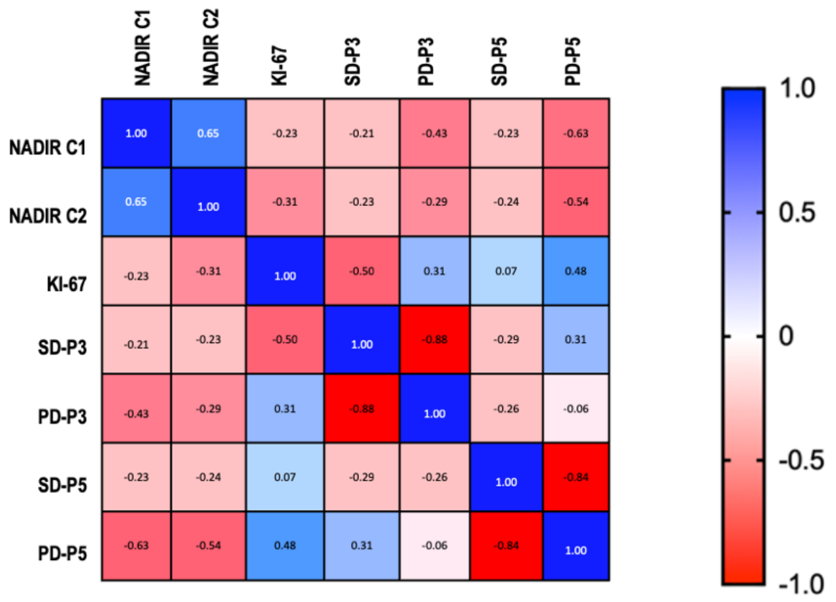

Table S1 : Primers used for mRNA and miRNA quantification

Primers	**Forward**	**Reverse**
miR-31	5’-AGGCAAGATGCTGGCATAGCT-3’	
miR-129-5p	5’-CTTTTTGCGGTCTGGGCTTGC-3’	
miR-133a	5’-AGCTGGTAAAATGGAACCAAAT-3’	
miR-215	5’-ATGACCTATGAATTGACAGAC-3’	
miR-183	5’-TATGGCACTGGTAGAATTCACT-3’	
miR-196b	5’-TAGGTAGTTTCATGTTGTTGGG-3’	
RNU6-1	5’-CTCGCTTCGGCAGCACA-3’	
GAPDH	5’-ACAGTCAGCCGCATCTTCTT-3’	5’-TTGATTTTGGAGGGATCTCG-3’
ATP	5’-GGCGCCTCTGTCATTCTACT-3’	5’-CAACAAATCTCGCGAATCCTC-3’
BRAF	5’-CTTTCCCCAAATTCTCGCCTC-3’	5’-GGACAGGAAACGCACCATA-3’
HDAC9	5’-GACGTGTGGTGTTGGCTCTA-3’	5’-GTGGCTCCAGCTCATTTCCT-3’
PANK2	5’-CGGCCGCTTTTTCCATGGTT-3’	5’-AAGCCACATTGGAGGTCAGG-3’
SSTR3	5’-AAGCGGTCTAGAAGTGGGTG-3’	5’-CAGCTATTTGCCTGCCCAT-3’
SSTR5	5’-TTCTTCTCTTGCAGAGCCTGAC-3’	5’-ATGTTGGTGACGGTCTTCATCT-3’
CXCL14	5’-GGACCCAAGATCCGCTACAG-3’	5’-CTTCGTAGACCCTGCGCTTC-3’
CDKN1B	5’-GGCAAGTACGAGTGGCAAGA-3’	5’-AGAAGAATCGTCGGTTGCAGG-3’
CDKN2A	5’-AGTCTGCAGTTAAGGGGGCA-3’	5’-ATCATCATGACCTGGTCTTCTAGG-3’
DDB2	5’-GGAGGGAACAACTAGGCTGC-3’	5’-TTCCAAAGCTCTTTGCCGTC-3’
XPC	5’-GAGCAAGGCCCGGCG-3’	5’-GAAAGTCCCTGAGGTCATCCC-3’
BAX	5’-GGAGCTGCAGAGGATGATTG-3’	5’-GGAGACAGGGACATCAGTCG-3’

Table S2: Univariate and multivariate Cox regression analysis of the 12 mRNA and the 6 miRNA associated with Recist

Gene	Univariate HR	Univariate pvalue	Multivariate HR	Multivariate pvalue
miR-31	2.32	<0.001	1.03	0.01
miR-129-5p	0.87	<0.001	0.88	0.08
miR-133a	1.32	<0.001	1.02	0.06
miR-215	1.12	<0.001	0.85	0.06
miR-183	1.14	<0.001	1.06	0.02
miR-196b	1.27	<0.001	1.06	0.01
ATP	1.11	<0.001	0.84	0.06
BRAF	1.51	<0.001	0.88	0.08
HDAC9	1.23	<0.001	1.02	0.06
PANK2	1.11	<0.001	0.83	0.09
SSTR3	1.65	<0.001	1.06	0.02
SSTR5	1.65	<0.001	1.07	0.02
CXCL14	1.18	<0.001	0.88	0.07
CDKN1B	1.11	<0.001	1.01	0.09
CDKN2A	1.11	<0.001	1.11	0.09
DDB2	1.58	<0.001	1.19	0.01
XPC	1.65	<0.001	1.12	0.001
BAX	1.59	<0.001	1.07	0.001
